# Random Forest Segregation of Drug Responses May Define Regions of Biological Significance

**DOI:** 10.3389/fncom.2016.00021

**Published:** 2016-03-09

**Authors:** Qasim Bukhari, David Borsook, Markus Rudin, Lino Becerra

**Affiliations:** ^1^Institute for Biomedical Engineering, ETH Zürich and University of ZürichZürich, Switzerland; ^2^Pain and Analgesia Imaging Neuroscience Group, Departments of Anesthesia, Perioperative and Pain Medicine, Boston Children's HospitalWaltham, MA, USA; ^3^Department of Radiology, Boston Children's HospitalWaltham, MA, USA; ^4^Institute of Pharmacology and Toxicology, University of ZürichZürich, Switzerland

**Keywords:** fMRI, random forest, machine learning, phMRI, pharmacology, buprenorphine

## Abstract

The ability to assess brain responses in unsupervised manner based on fMRI measure has remained a challenge. Here we have applied the Random Forest (RF) method to detect differences in the pharmacological MRI (phMRI) response in rats to treatment with an analgesic drug (buprenorphine) as compared to control (saline). Three groups of animals were studied: two groups treated with different doses of the opioid buprenorphine, low (LD), and high dose (HD), and one receiving saline. PhMRI responses were evaluated in 45 brain regions and RF analysis was applied to allocate rats to the individual treatment groups. RF analysis was able to identify drug effects based on differential phMRI responses in the hippocampus, amygdala, nucleus accumbens, superior colliculus, and the lateral and posterior thalamus for drug vs. saline. These structures have high levels of mu opioid receptors. In addition these regions are involved in aversive signaling, which is inhibited by mu opioids. The results demonstrate that buprenorphine mediated phMRI responses comprise characteristic features that allow a supervised differentiation from placebo treated rats as well as the proper allocation to the respective drug dose group using the RF method, a method that has been successfully applied in clinical studies.

## Introduction

Optimal dosing is an important process in the evaluation or development of pharmaceutical agents. For CNS drugs, parameters evaluated comprise pharmacokinetic readouts such as drug penetration through the blood-brain barrier (Alavijeh et al., 2005), receptor binding, or analysis of drug concentration in cerebro-spinal fluid (CSF; Friden et al., 2010). Some of them rely on invasive procedures and are therefore of limited clinical use. In addition, they do not provide information on pharmacodynamic efficacy. Alternatively, drug dosing may be based on assessing pharmacodynamic responses, which for neuroactive drugs may include the analysis of effects on brain circuits using objective readouts such as functional magnetic resonance imaging (fMRI).

fMRI responses constitute an objective measure that can be used in disease diagnosis, prognosis, and evaluation of treatment effects (Borsook et al., 2011a,b, 2012). Yet, fMRI response patterns are complex and often difficult to analyze. In recent years, machine learning and pattern recognition have entered the field of neuroimaging (Formisano et al., 2008) based on their ability of detecting subtle, non-strictly localized effects, that commonly would escape univariate statistical analyses. Machine learning tools enable pattern recognition algorithms to uncover a functional relationship among the brain response patterns, in particular by identifying features that allow classification into different groups for diagnostic purposes, prognosis, or for the analysis of therapy responses (Strimbu and Tavel, 2010).

Most analytical approaches concentrate on obtaining general, population-based results. While such analyses are important, methods that allow proper allocation of individual patients to the respective groups are of critical importance for diagnostic purposes. Several approaches are available to solve this problem; using support vector machines (e.g., generative embedding Brodersen et al., 2014) or other machine learning techniques (Schrouff et al., 2013). These techniques, however, do not discriminate the feature vectors based on their importance of classification.

Here, we use Random Forest (RF) as a means of identifying brain regions that display differential responses under different pharmacological conditions (high and low doses of buprenorphine and saline), which should be suited for diagnosis at the level of the individual patient providing classification probabilities. RF is based on combining two independent ideas of random selection of features and bagging to construct decision trees with controlled variance. It has been used increasingly in medicine (Ghose et al., 2013; Casanova et al., 2014; Simonsen et al., 2014). A big advantage of using RF is that it adds a confidence label to the classification due to its probabilistic nature. This is not the case for many other classification algorithms including SVM: even though SVM has been repeatedly used as classification tool in neuroimaging studies, it does not provide a probabilistic classification. As a consequence SVM may add labels to a sample even if it is unable to properly classify it. In contrast, upon using RF such samples might be identified based on their probability values of 0.5 (50%), and thus be labeled correspondingly (e.g., as “unclassified”).

RF allows for estimating the importance of feature vectors that are used for its classification, thereby providing information regarding the biological basis of the classification results. As RF also generates probabilistic results, it yields a measure of confidence in the classification results obtained (Disanto and Wiehe, 2013). The methodology described in that paper is unsupervised and thus suited for analysis of experimental data, for which modeling the experimental paradigm into the analysis is difficult or not possible. This is also the case for the study presented here given the temporal fMRI response to the administration of the drug is not known. However, the method is not restricted to conditions lacking a model description since differences in brain regions are calculated from the data and depend on the power contained within the group to differentiate them.

The goal of this study was to develop a methodological formulation based on RF to identify differences in fMRI responses in a region-specific manner in groups treated with the drug buprenorphine at different doses and a control (saline) group. We have previously reported the pharmacological effects of buprenorphine vs. saline using fMRI evaluation of the drug in rats (Becerra et al., 2013). Buprenorphine is a semisynthetic opioid compound with μ and κ receptor affinity that has been used in the treatment of opioid-dependent patients (Mattick et al., 2014) as well as for treatment of pain patients (Cote and Montgomery, 2014). The drug was shown to have similar analgesic effects in rodents and hence, is therefore well-suited for evaluating a classification scheme that would differentiate drug treatment from controls, as well as potentially discriminate different drug doses.

## Methods

### Imaging

The study was approved by the Massachusetts General Hospital's Animal Care and Use Committee. Male Sprague-Dawley rats (~300 g) were used for these experiments with 12 animals injected with 0.04 mg/kg (low dose; LD), 12 with 0.1 mg/kg (high dose; HD) buprenorphine and 13 (controls) with saline. Solutions were prepared to have a 1 ml/kg concentration, for saline a 1 ml/kg infusion was administered. For imaging, anesthesia was induced with 3% isoflurane for 15 min and the rats were positioned in the MRI cradle. A tail vain was placed for drug infusion. The infusion scan lasted 25 min; after 5 min of baseline scanning, the drug/saline was infused over a period of 2 min. fMRI data were acquired using a 4.7 T Biospec scanner (Bruker Biospin Ltd, Billerica) with a surface coli for transmit/receive. An EPI sequence with TR/TE = 2.5 s/11 ms was used, with 12 slices (1.5 mm thick, FOV = 3.0 cm, matrix = 64 × 64) recorded. 600 volumes were acquired resulting in an acquisition time of 25 min. A short TE was used to reduce susceptibility artifacts while maintaining sufficient contrast. For detailed information the reader is referred to Becerra et al. (2013).

### Analysis

**Figure 1:**
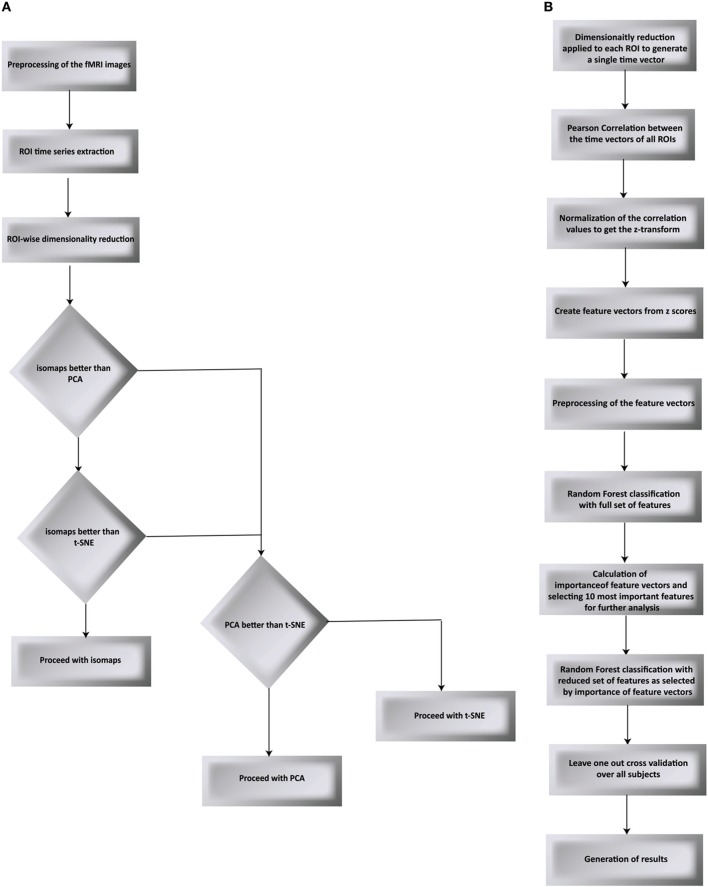
**Flow chart of the proposed processing pipeline**. **(A)** compares PCA, t-SNE, and isomaps to find the best suited dimensionality reduction for our experiment. The decision was taken by testing for classification and validated using LOO validation **(B)** goes on to apply Random Forest as the classification algorithm followed by the LOO validation.

We have proposed a novel approach in this work to find the brain regions that differentiate between two different drug states. The pipeline we have proposed is shown in Figures 1A,B and is discussed below. We used RF to differentiate between the drug groups and further identify the most important feature components that gives us the brain regions that differ between the groups. The steps below describe each processing step that we applied over the data.

#### Pre-processing

Pre-processing was carried out utilizing FSL tools (Jenkinson et al., 2012) adapted for rat brain anatomy and included motion correction and spatial smoothing (0.7 mm). No high pass filtering was applied. Brain extraction was performed using in-house software. Functional data were registered to an in-house atlas for group analysis. A Gaussian smoothing kernel for functional volume and reference volume was used.

Region of Interest (ROI) time series extraction: ROIs across the whole brain were extracted based on our internal MRI atlas developed from a histological one (Paxinos, 2007). In total, 45 structures were used to extract time series.

#### Dimensionality reduction

**Figure 2:**
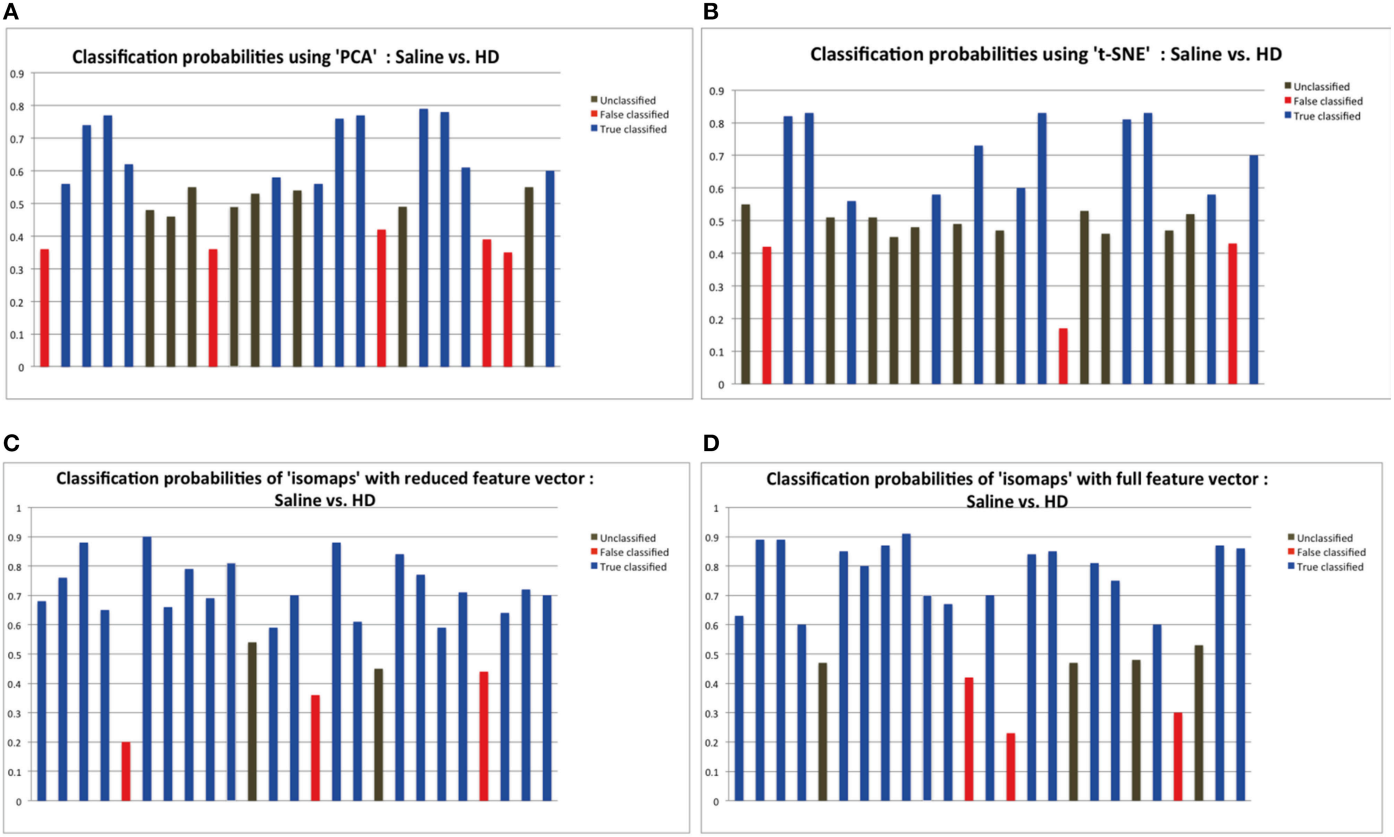
**The probabilities of classification results**. Data below 0.45 probabilities presents a false result, while anything greater than 0.55 probability presents the correct result. The classification probabilities between 0.45 and 0.55 were considered “unclassified” because of the uncertainty in classification results. Dimensionality reduction using isomaps clearly presents better results that PCA and t-SNE method as shown by the experiments. Probabilities shown here are the result of applying random forest for classification using the specific dimensionality reduction technique over the individual ROIs. **(A)** Probabilities showing 11 correct classification using PCA as the dimensionality reduction method for the individual ROIs. **(B)** Probabilities showing 10 correct classification using t-SNE. **(C)** Probabilities showing 20 correct classification using isomaps. **(D)** Comparison between full and reduction feature set for prediction: Classification probabilities and the predictions using the reduced feature set, that is only from the most important 6 features as obtained from the Random Forest variable importance graph.

Time series from predefined ROIs were extracted from the pre-processed data. In order to feed these time series into a machine-learning algorithm, it was necessary to reduce the dimension of data in an efficient way. We used isomaps as dimensionality reduction technique after having carried out a comparison analysis with Locally-Linear Embedding (LLE), t-Distributed Stochastic Neighbor Embedding (t-SNE), and PCA using DELFT implementations. The reduced time data set for a ROI was then correlated with that of other ROIs to form a z-correlation matrix. Random forest algorithm was applied to the z-correlation maps of the unlabeled data set to identify whether there was enough power contained in the dataset to classify the groups correctly. Isomaps generated the lowest false classification probabilities when used as the dimensionality reduction technique (Figure 2). It is important to realize that the result of dimensionality reduction is dependent on the classification result and vice versa.

#### Preprocessing of the feature vectors

After the dimensionality reduction, we applied a high pass filter to smooth the data. Since the data had a lot of undesired small fluctuations, high pass filtering gave us a better model of the time series data contained in every region. We also normalized each feature (column) to have mean 0 and standard deviation 1.

#### Pearson correlations across all regions and normalization of values

Pearson correlation between ROIs was carried out using the dimensionally reduced vectors to determine a full connectivity map between all the regions. Connection strengths between two ROIs are expressed by:

$$\begin{array}{l} \rho X,Y=corr\left(X,Y\right)\\ \;=\frac{E[(X-\mu X)(Y-\mu Y)]}{\sigma X\sigma Y} \end{array}$$

Where *X* = *X*(t) and *Y* = *Y*(t) represent the two time series corresponding to the two ROIs. The correlation values were then fitted to a Gaussian curve adopting the Fisher z-transformation with the following formula:

$$\begin{array}{l} z_{X,Y}=0.5\cdot \text{log}\left[\left(1+\rho_{X,Y}\right)∕\left(1-\rho_{X,Y}\right)\right] \end{array}$$

#### Conversion of a matrix in to a vector

The *z*-scores from the Pearson correlation were passed through another pre-processing step. We sequentially converted the complete matrix in to a 1D vector, keeping the record of its dimensions so at any time the backtracking can result in the actual brain regions and voxel that are of interest.

#### Classification of groups by random forest using the generated feature vectors

Classification of the groups was done using Random Forest. RF is based on the principle of aggregating several binary decision trees built on several bootstrap samples drawn uniformly from the learning set. The aggregate of all the tree classifiers constitute the final prediction of the Random Forest. Since each tree predicts a class, a confidence interval is generated that described the percentage of votes for either class.

Feature vectors comprising the connectivity values across all brain regions were used as input for the classification. In order to evaluate the performance of the classification tool, we used the “leave-one-out” (LOO) cross validation technique (Lachenbruch and Mickey, 1968; Luntz and Brailovsky, 1969), thereby each and every sample was tested with regard to the classification in an unbiased fashion. LOO requires the classification algorithm to run N times, leaving one of the subjects each time out, treating it as a test subject and training the classifier on N–1 other subjects. This assures that each and every of the subject is tested and the results neither contain any bias nor any chance sampling. Prediction error and variable importance was estimated from the “out-of-bag” sample of observations.

#### Calculation of importance of feature vectors

We used the RF library from R (Liaw and Wiener, 2002) to calculate the importance of feature vectors in order to find the most important feature vectors for successful classification of groups. This provides a variable importance index for feature vectors using RF permutation index as the indicator.

RF calculates variable importance by estimating out of bag (OOB) error, which is the proportion of misclassified data. For each OOB sample we permute at random the *i*-th variable values of the data. The variable importance of the *i*-th variable is the mean increase of the error of a tree. The higher the value, the more important is the variable. The Supplementary Figure 1 shows the importance of variables graph in a decreasing order. It is clear, that first few features in the graph carry more information than the rest. To keep the uniformity among the experiments, we selected 10 most important features for further processing. The other features carry similar and smaller information contents as shown in the figure. As an additional robustness check, we ran the algorithm with features from 11 to 990, however as can be seen from Supplementary Figure 2, the prediction accuracy is decreased. This indicates that the most important information was contained in the 10 most important features as calculated by variable importance.

The most important feature vectors for LD vs. HD group were selected based on the analysis of the other two groups that are Saline vs. LD and Saline vs. HD. The uncommon correlation pairs between these two groups were selected as the features of interest.

#### Re-evaluation of classification to verify the power contained in the feature vectors

In order to validate the feature vectors obtained through variable importance index, we classified the groups again but this time with a reduced set of features that were the top important features as selected by the R library using the variable importance function. We restricted ourselves to select 10 most important features out of 990 total features in each case. Dimensionality reduction, important feature selection and final classification were all done inside the LOO framework.

## Results

Figure 2 depicts the classification results as a function of the dimensionality reduction approach used: PCA, t-SNE, and Isomaps. Shown are the probabilities that the individual animals are correctly attributed to its group be it saline (control) or high-dose (HD) group of buprenorphine treatment. Using Isomaps as dimensionality reducing technique generated the highest correct classification probabilities (*N* = 20) as compared to PCA (*N* = 12) and t-SNE (*N* = 11). Hence, Isomaps was used as the method of dimensionality reduction for the whole study.

Though the classification was successful, we still needed to find the most important features (regional connectivities) that made this classification possible. This is illustrated in Figure 2D depicting the prediction results of a set of selected brain regions as indicated from RF variable importance for the comparison control vs. LD. Figure 2C should be compared with Figure 2D, which shows the analogous analysis for ROIs across the whole brain. The results indicate that using specific but more informative regions preserves the classification result, and thus proves the concept that these regions contain most of the useful information for the classification between the two groups. Classification accuracy was evaluated using the LOO method (Table 1).

**Table 1:** **Classification accuracy based on leave one out cross validation with all 45 regions (990 features) considered for the classification**.

	**Average correctly predicted in k times leave one out validation**	**Average false predicted in k times leave one out validation**	**Average unclassified in k times leave one out validation**	**Total number of subjects “k”**
Saline vs. LD	18	4	3	25
Saline vs. HD	18	3	4	25
LD vs. HD	6	9	9	24

Similar analyses have been carried out for the HD group. Classification was first applied with the complete feature set (990 features), followed by the calculation of important features. These important features were then used for re-classification. The accuracy of the classification procedure was evaluated using the LOO method. Reducing the number of feature vectors to include the 10 most important ones preserves the classification accuracy, proving that the most important information lies in the selected feature vectors (Table 2). When comparing LD vs. HD, the initial classification using all 990 features with leave one out validation generated only chance probability. Thus, the lack of significant result also prohibited us from further continuing the analysis to find the most important features for classification. To solve this problem we used the mutually exclusive method from sets, i.e., we selected the anatomical regions which were found among the most important features of Saline vs. LD and Saline vs. HD comparisons, however selected only those anatomical regions present in one of the two comparisons only. The rationale behind was if it exists in only one of the comparisons, it is more likely to be the effect of the dose rather than the saline or other mutual effects in the comparison. Once these uncommon correlation pairs between these two groups were selected as features of interest, we applied the classification algorithm over the reduced feature set as selected from this method, and applied LOO cross-validation to obtain classification accuracy of 66.6%. While this work-around yielded some reasonable classification results, the results need to be handled with care.

**Table 2:** **Classification accuracy based on leave one out cross validation after selecting the top 10 features from the variable importance as indicated by Random Forest**.

	**Average correctly predicted in k times leave one out validation**	**Average false predicted in k times leave one out validation**	**Average unclassified in k times leave one out validation**	**Total number of subjects “k”**
Saline vs. LD	20	2	3	25
Saline vs. HD	20	3	2	25
LD vs. HD	16	6	2	24

Table 3 indicates the brain structures that anchor the classification using the reduced set of features. Common structures that discriminate fMRI response of the three treatment groups included thalamus, hypothalamus, hippocampus, caudate putamen, and colliculus. Only the 10 most important features in the classification are listed, while few extra regions are also listed with their rank among importance of feature vectors, to provide better comparison between Saline vs. LD and Saline vs. HD analysis.

**Table 3:** **Anatomical structures found important for the classification**.

**Cortical structures**	**Regions**	**Sides**	**Saline vs. LD**	**Saline vs. HD**	**LD vs. HD**
Sensorimotor cortex	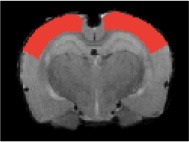	Right	2.76	2.28	
		Left	<2	<2	
Anterior cingulate cortex	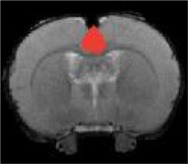		2.21	2.13	^*^
Entorhinal cortex	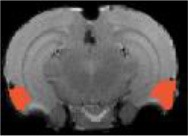	Right	2.03	2.21	
		Left	<2	<2	
Insula	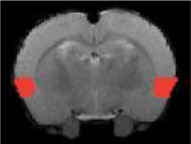	Right	2.09	<2	
		Left	2.05	<2	^*^
Hippocampus	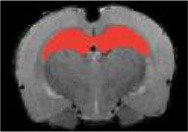	Right	2.21	<2	
		Left	<2	2.91	
**Subcortical structures**	**Regions**	**Sides**	**Saline vs. LD**	**Saline vs. HD**	**LD vs. HD**
Thalamus ventral	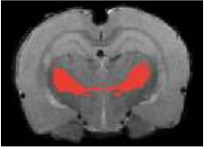	Right	5.3	3.87	
		Left	4.6	3.23	
Thalamus posterior	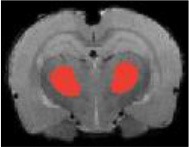	Right	<2	3.57	
		Left	<2	2.09	^*^
Hypothalamus	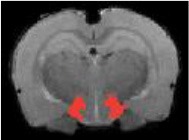	Right	2.9	3.87	
		Left	1.95	2.41	
Cudate Putamen	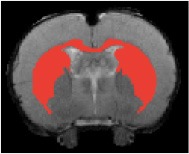	Right	2.09	2.96	
		Left	<2	2.13	^*^
Amygdala basal lateral	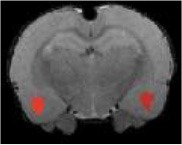	Right	2.87	2.34	
		Left	2.86	2.23	
Amygdala anterior	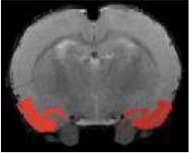	Right	2.07	2.27	
		Left	<2	<2	
Nucleus accumbens	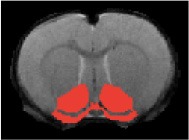	Right	<2	2.28	^*^
		Left	2.86	3.57	
Superior colliculus	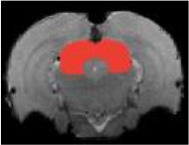	Right	<2	2.53	^*^
		Left	<2	2.53	^*^
Inferior colliculus	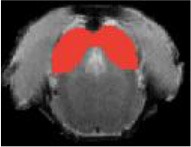	Right	<2	2.97	^*^
		Left	<2	2.97	^*^

The table below shows the cortical and subcortical structures with the individual importance value for each classification.

For Saline vs. LD and Saline vs. HD, only those values are shown that are greater than 2. For LD vs. HD, the regions that were found important for the classification are marked with asterisk (^*^).

## Discussion

While classification using machine learning approaches have been used for pain states on the basis of fMRI data, the approach has been hardly applied for evaluating drug efficacy (Salat and Salat, 2013). Here, we have used RF for identifying brain regions displaying differential responses in response to treatment with the opioid drug buprenorphine and saline in a supervised manner. A critical step in preprocessing data for the machine-learning tool is dimensionality reduction based on the experimental data. We have found that isomaps yields better classification accuracy compared to PCA or t-SNE based reduction methods. A feature of Isomaps is that it considers voxels in their context by intrinsic construction of a neighborhood graph based on the geodesic distance. Such neighborhood relationships are important factors when analyzing brain functional data, as local networks of connected voxels make up a functional region. This might explain why isomaps outperformed other classifiers in our case.

For the classification, we choose the RF classifier, since based on theoretical arguments it should yield optimal classification performance in the sample limit, which has been successfully demonstrated to be the case for many biomedical classification problems (Statnikov and Aliferis, 2007). The explanation of the good performance of RF is related to the good quality of each regression tree (Breiman, 2001). One of the big advantages that RF offers is that it automatically saves the features that are most critical for the classification purpose: these features are related to a relatively small number of anatomical regions that apparently play an important role in capturing treatment response. It is to our knowledge the first time that such method has been applied for analysis of fMRI response to pharmacological stimulation.

Brain region displaying a differential response in buprenorphine treated rats at either dose compared to saline treated controls are listed in Table 2. Regions identified for both drug doses upon comparison with saline (HD vs. Saline; LD vs. Saline) have been reported to process pain (Tracey, 2008). Additional regions were identified in both groups such as the caudate putamen, superior, and inferior colliculus. Interestingly, all regions identified for the LD buprenorphine group were also found in the HD group, though not necessarily in the same rank order. Regions that show differential responses depending on the buprenorphine dose were amygdala, hypothalamus, nucleus accumbens, posterior thalamus, and sensorimotor, insular, and entorhinal cortices. Many of these areas appear as classifiers either in HD vs. saline or LD vs. Saline. The relevance of the classification results is also supported by the notion that essentially all the structures important for classification display high levels of mu opioid receptors. In addition they are involved in aversive processing, which might be inhibited by opioids.

In Becerra et al. (2013), cingulate cortex, insula, cerebellum, and thalamus had been shown as important anatomical regions that differentiate between LD vs. HD group using model based analysis (GLM) and our data largely agree with their findings. However in our results, insula appears in LD vs. Saline and LD vs. HD comparisons but not in HD vs. Saline comparison. Amygdala was not reported in the GLM paper, however it appears as an important region in our results. Model based approaches are limited by the intrinsic nature of the model: i.e., features not comprised in the model cannot be extracted from experimental data. However model-free approach such as the RF classification is not based on the correspondence of the basic assumptions with the actual experimental data. Being data driven it just searches for differences in the responses among two (or more groups) irrespective of their actual shape, and thus might identify regions that are not detected with GLM or related methods.

Brain regions of high discriminative power for all three comparisons (LD vs. saline, HD, vs. saline, LD vs. HD) were hippocampus and thalamus, while insula, posterior thalamus, amygdala differentiated the LD from the HD group. What may be important in the functionality of these regions that contribute to the differentiation process? There may be a number of interrelated processes including (1) opioid receptor numbers, (2) function of specific regions in endogenous pain control, and (3) connectivity between these regions contributing to whole brain “interrogation” of the three conditions.

Buprenorphine is known to bind to the μ- and κ opioid receptors (Becerra et al., 2013), the nociceptin/orphanin receptor (Khroyan et al., 2015), and the opioid receptor like (ORL-1) receptor (Lutfy and Cowan, 2004). The μ-opioid receptors occur with high concentration in cerebral cortex, thalamus, striatum (striosomes), amygdala, periaqueductal gray (Mansour et al., 1987), while the κ-opioid receptors are found in hypothalamus and also periaqueductal gray. In general the distribution profiles of the primary opioid receptors—μ, κ, δ—are slightly different but also share significant overlap in structures including the amygdala and hippocampus. High levels of nociceptin receptor occur in cortex, hippocampus (dentate gyrus), amygdala, hypothalamus, and septal nuclei (Houtani et al., 2000), while ORL-1 receptor is found predominantly in cortical areas, olfactory regions, limbic structures, and thalamus (Mollereau and Mouledous, 2000). The regions identified in the RF signature discriminating drug effects from vehicles are essentially those outlined by this receptor distribution (Table 2).

While it is unclear how the specific regions contribute as a result of their own primary function (e.g., anterior insula and awareness) or interactions as a result of buprenorphine-receptor activation of neurons with efferent projections, the drug must produce alterations in brain circuits that differentiate the three conditions. The amygdala is involved in analgesia, emotion, and also decision-making (LeDoux, 2007). Of the various structures identified, amygdala has probably has the highest binding of all three receptor targets of buprenorphine and it is therefore not surprising that it shows a strong response to the drug as compared to saline. The hippocampus known to be involved in memory formation, spatial orientation and pain modulation, also participates in the stress response (Gray et al., 2014). This latter response can be diminished by opioids, presumably due to its μ receptor effects (Okutani et al., 1989), which may explain its prominent role as classifier in discriminating the response to drug treatment as compared to controls. Nevertheless, it should be remembered that buprenorphine interacts with several opioid and opioid-like receptors systems, the effects of which might be even counteracting (e.g., ORL-1 activity vs. μ receptor effects). Hence, interpreting the occurrence of specific brain areas in the discriminative feature vectors in terms of cognitive and emotional effects remains speculative, in particular when dealing with anesthetized animals as anesthesia may further modulate the fMRI responses to drug administration. On the other hand it is reassuring that regions associated with nociceptive processing clearly show up, indicative of the analgesic activity of the drug.

While RF yielded reasonable classification results regarding the nature of structures identified, we observed that several of the important regions appear unilaterally only. Given the nature of the condition, i.e., a pharmacological stimulus with a systemically administered drug, and the more or less symmetrical distribution of its molecular targets across the brain, on would have expected that feature vectors display bilaterally symmetry. The question then arises whether this left/right asymmetry is of biological origin or whether it is an artifact of the analysis. Finding the most relevant features involves inherently a ranking and when maintaining of fixed number of “most important feature vectors” also a thresholding. As a consequence, it is well conceivable that a structure within one hemisphere may not pass this threshold, leading to an apparent laterality. Obviously, this may be accounted for by relaxing the threshold criterion, i.e., by maintaining bilaterality if the difference between the two hemispheres is within “the noise range.” This also became obvious, when analyzing the dose dependence of the buprenorphine response. When limiting the number of feature vectors in the analysis to 10, there was the counter-intuitive result that features discriminating drug from saline appeared in the LD but not in the HD group. However, all LD features were contained in the 17 most important features of the HD group indicating that when analyzing the data it is important not to just be restricted to a fixed number of features.

The reasons underlying the failure of typical classification between LD vs. HD can be explained in several ways. LD and HD doses were selected based on publications indicating minimal and significant analgesic effects on behavioral measures in rats. Discrepancies between behavioral outcome and imaging findings are not unusual, as the former depend on the specific test paradigm applied and may be confounded by processes such as learned behavior or reflexive responses. In contrast imaging responses depend on the physiological baseline state, which is affected by the use of anesthesia. Other factors that might explain the lack of a difference between the LD and HD group might arise from inter-individual differences in the bioavailability of the drug, which would result in larger variability reducing the statistical power in discriminating the two states. Finally there might be a ceiling effect regarding the fMRI response. Future studies using expanded dose ranges and larger cohorts should clarify this aspect.

## Conclusion

We have used RF to classify rats based on the fMRI signature in response to systemic administration of buprenorphine at to different doses or saline to rats. The regions that turned out to be most important for the proper classification of animals were those displaying high levels of opioid and opioid-like receptors known to be the buprenorphine target, in particular structures associated with nociceptive processing, but also the limbic system. Using the LOO approach, the classification accuracy was 80% for the comparison of drug vs. placebo, while there were 66% of correct assignments for the comparison LD vs. HD. RF appears an attractive machine-learning tool suited for classification of individuals based on their response to a neuroactive compound.

## Author contributions

QB: analyze, write manuscript. MR: write manuscript. DB design, write manuscript. LB: design, acquire data, analyze, and write manuscript.

### Conflict of interest statement

The authors declare that the research was conducted in the absence of any commercial or financial relationships that could be construed as a potential conflict of interest.
