# Supplementary material for: Random Forest Segregation of Drug Responses May Define Regions of Biological Significance
Source: Front Comput Neurosci. 2016 Mar 9;10:21. doi: 10.3389/fncom.2016.00021 (PMC4783407; doi:10.3389/fncom.2016.00021)
Supplement: Supplementary file 1 [file DataSheet1.DOCX]

Supplementary Material

Random Forest Segregation of Drug Responses May define Regions of Biological Significance

Bukhari Q^1^, Borsook D^2^, Rudin M^1,3^, and Becerra L^2^*

*** Correspondence:** Becerra L: lino.becerra@childrens.harvard.edu

# Supplementary Data

Following supplementary material has been included in the manuscript.

# Supplementary Figures and Tables

##
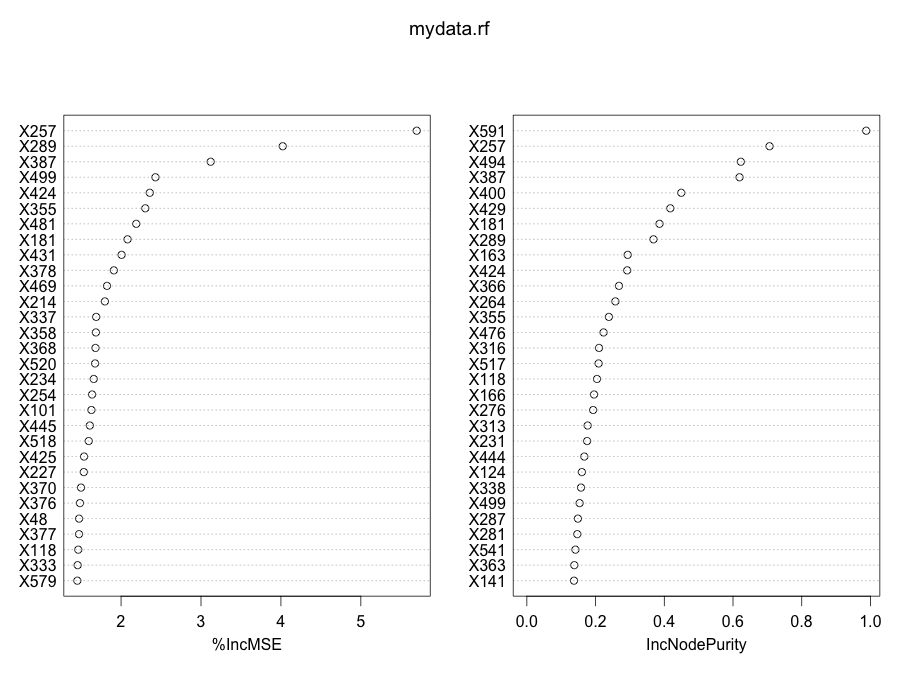
Supplementary Figures

**Supplementary Figure 1.** The figure shows the variable importance map with the top features representing more information than the rest.


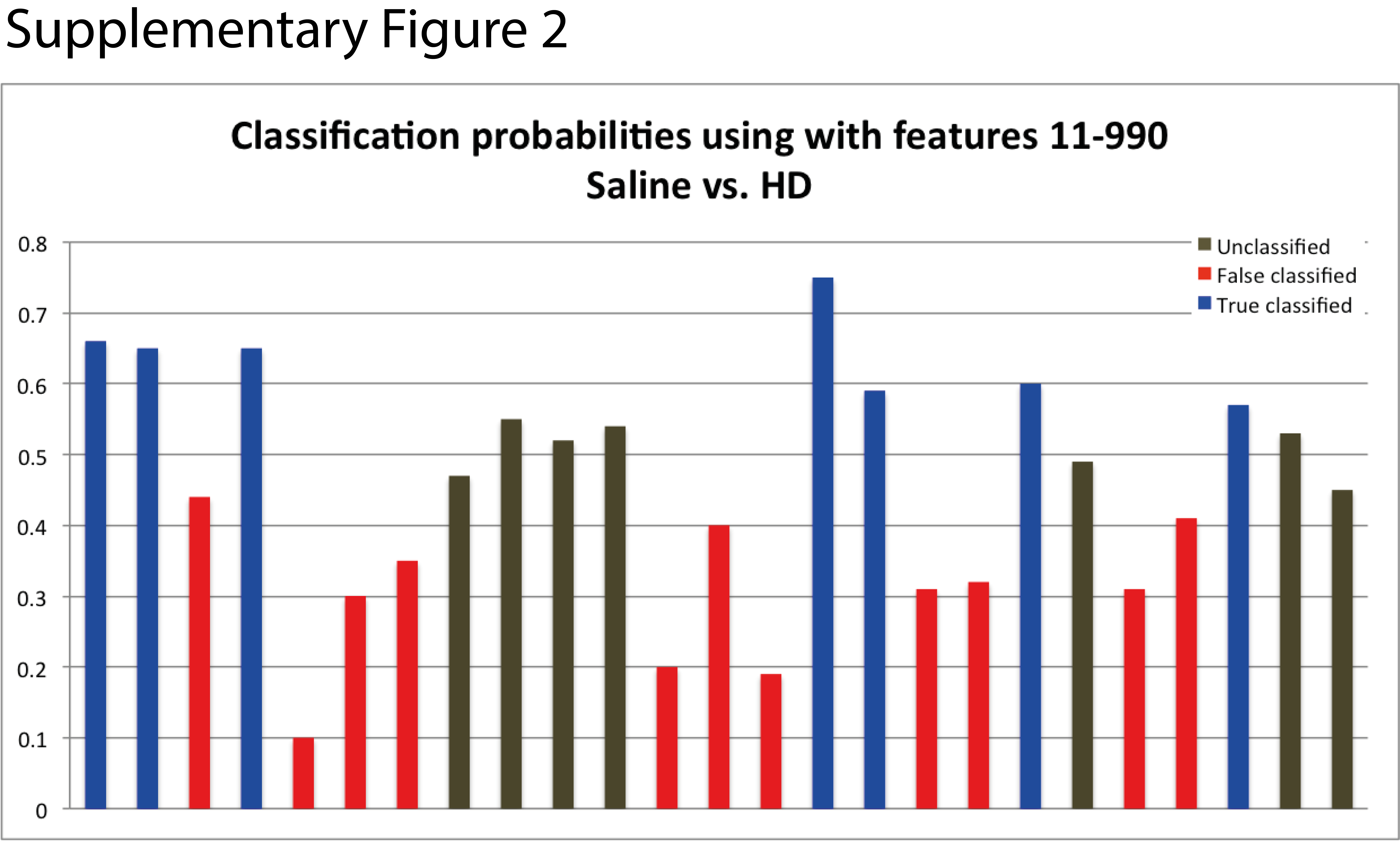


**Supplementary Figure 2.** The figure shows the classification results for Saline vs. HD with features 11 to 990 in the decreasing order of their importance values. The classification results with LOO show that the prediction accuracy with top 10 important features is greater than prediction accuracy with features 11-990
