# Supplementary figures and images for: Random Forest Segregation of Drug Responses May Define Regions of Biological Significance
Source: Front Comput Neurosci. 2016 Mar 9;10:21. doi: 10.3389/fncom.2016.00021 (PMC4783407; doi:10.3389/fncom.2016.00021)

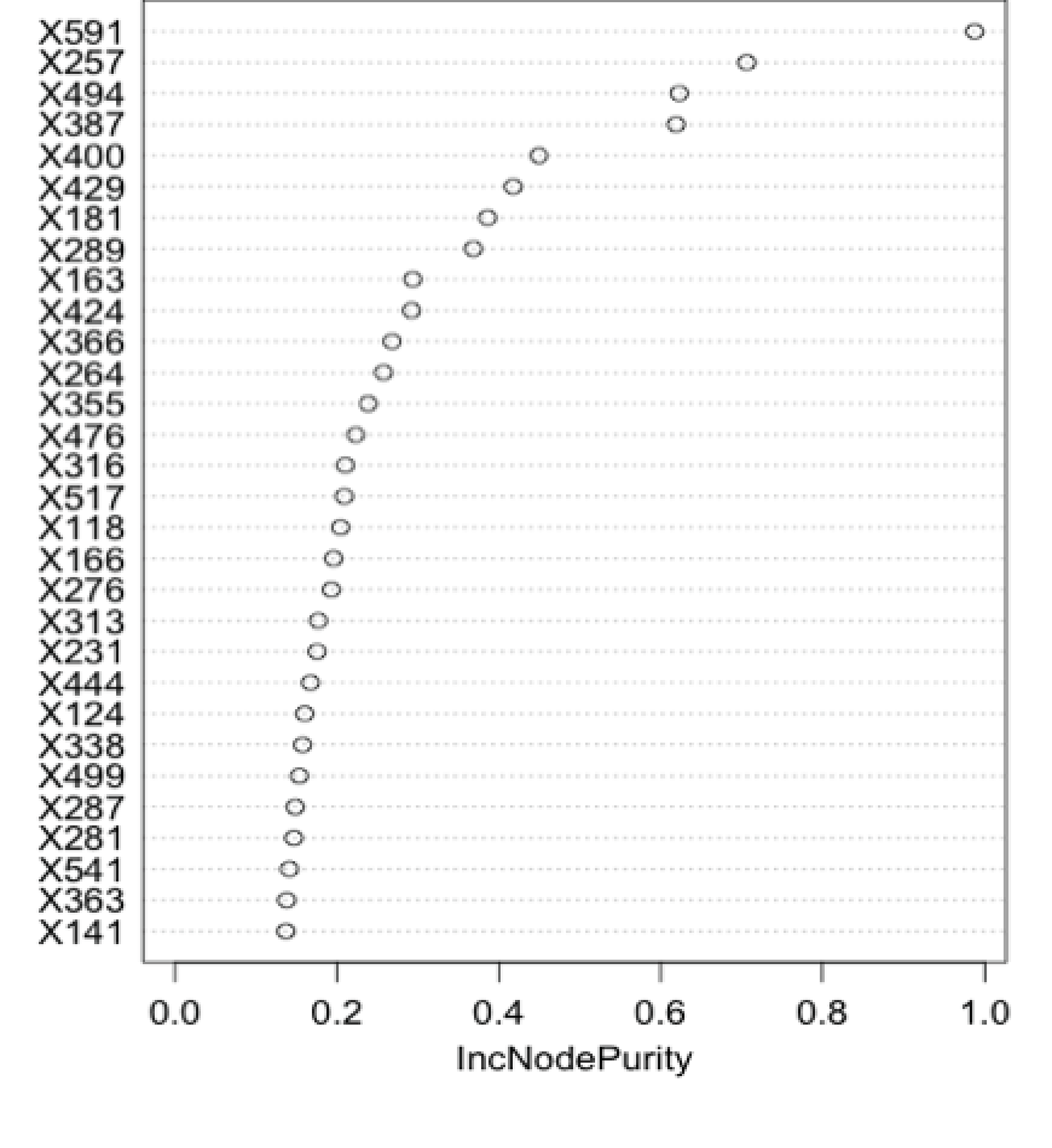

Supplement: Supplementary file 2 [file Image1.TIF]

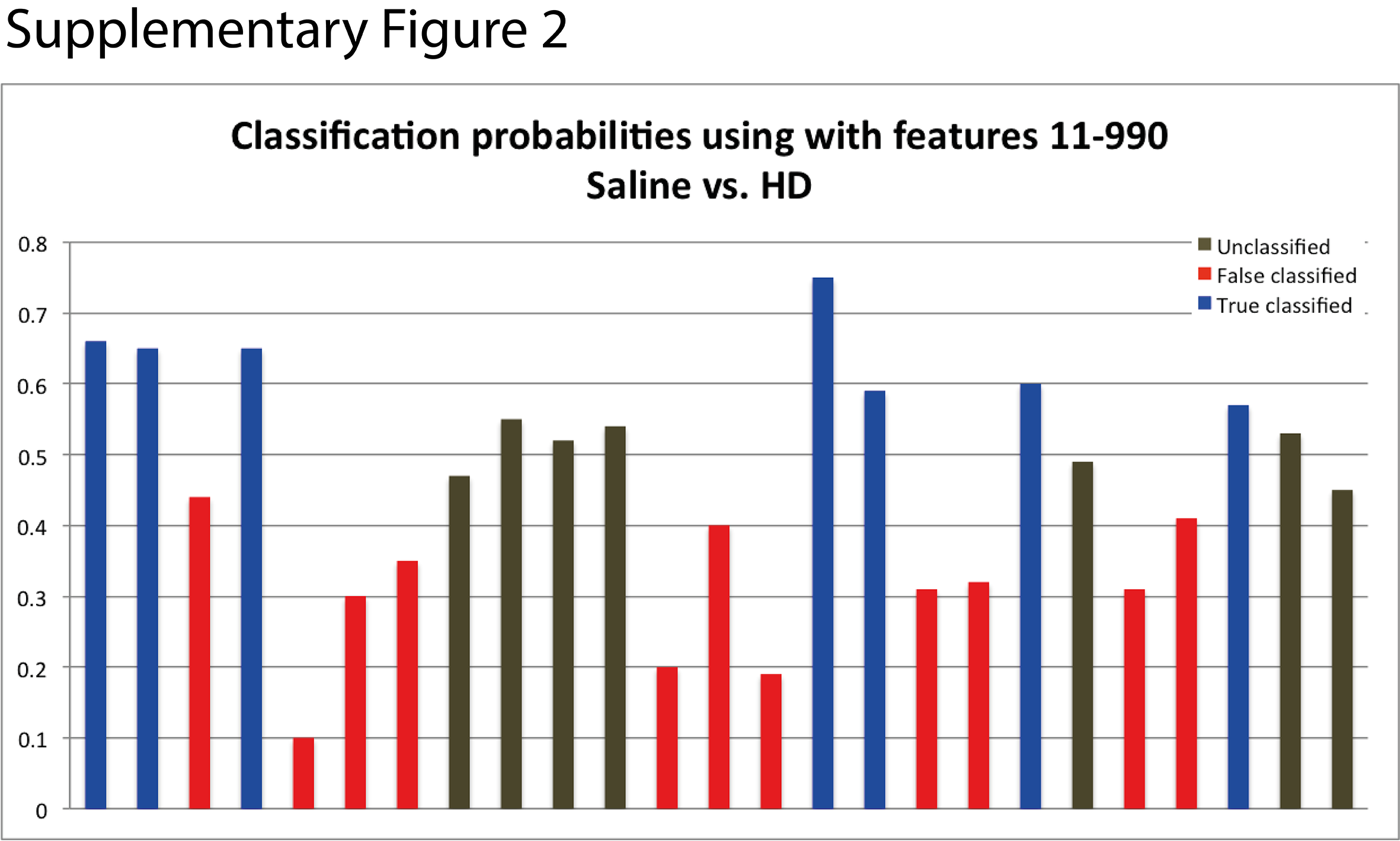

Supplement: Supplementary file 3 [file Image2.TIF]
